# GWO-Optimized BPNN for Abrasion Resistance Prediction of Nano-SiO_2_ and Hybrid Fiber Reinforced Geopolymer Gel Concrete

**DOI:** 10.3390/gels12060463

**Published:** 2026-05-25

**Authors:** Jiawei Han, Peng Zhang, Xiaobing Dai, Canhua Lai

**Affiliations:** School of Water Conservancy and Transportation, Zhengzhou University, Zhengzhou 450001, China; hanjiawei@stu.zzu.edu.cn (J.H.); daixb@zzu.edu.cn (X.D.); 13395998071@163.com (C.L.)

**Keywords:** nano-SiO_2_, steel-PVA hybrid fiber, abrasion resistance strength, abrasion rate, gray wolf algorithm, mix design

## Abstract

Geopolymer gel concrete (GPC) is a kind of environmentally friendly concrete, which has become a potential alternative material to replace ordinary concrete. Traditional mix design of GPC is carried out under experimental conditions, which is time-consuming and labor-intensive. Geopolymer concrete (GPC) is intended for use in hydraulic structures, which are often exposed to water environments. Water flow exerts significant abrasion and erosion on these structures. If the abrasion resistance (AR) of the material is poor, the service life and service quality of hydraulic structures will be substantially reduced under the action of water flow. Therefore, AR is a key performance indicator for GPC in hydraulic engineering applications. This abrasion resistance can be enhanced by using fibers (for example, steel fibers, polyvinyl alcohol (PVA) fibers, and basalt fibers) and nanomaterials. Furthermore, there is a complex nonlinear relationship between the proportions of fibers and nanoparticles added and the properties of GPC. In this study, the circular ring test method and the underwater steel ball test method were conducted to investigate the AR of nano-SiO_2_ (NS) and hybrid fiber (NHF) reinforced geopolymer gel concrete (NHF-GPC). A backpropagation (BP) neural network (BPNN) model optimized by the Grey Wolf Optimizer (GWO) (GWO-BPNN) is established to predict the abrasion resistance strength (ARS) and the abrasion rate of NHF-GPC based on the circular ring test method. In addition, the ARS, abrasion rate, and average abrasion depth (AAD) based on the underwater steel ball test method were also predicted. The results indicate that the GWO-BPNN model demonstrates superior performance over the standard BPNN, exhibiting higher prediction accuracy, better fitting performance, and faster convergence speed. Specifically, for the circular ring test method abrasion rate prediction, GWO-BPNN reduced the root mean square error (*RMSE*) by 30.3% and lowered the mean absolute percentage error (*MAPE*) to 8.4%. The GWO-BPNN model established in this study can provide efficient and reliable theoretical support for the optimization of the NHF-GPC mix design.

## 1. Introduction

Global carbon emission lead to severe consequences such as climate change and ecosystem degradation. The cement industry is identified as a major carbon source. For each ton of cement produced, about 900 kg of carbon dioxide are emitted, which accounts for 5–7% of global carbon emissions [1,2,3]. Therefore, finding low-carbon cementitious materials is particularly important. Geopolymer gels, first identified and named by Davidovits in the 1970s [4,5], are a new type of green cementitious gel material. Geopolymer gel concrete (GPC), produced mainly from industrial by-products, is increasingly regarded as a viable replacement for traditional concrete [6]. Geopolymer gels possess many remarkable properties, including chemical corrosion resistance, high-temperature tolerance, and good durability. In addition, their preparation process can also reduce carbon emissions. Zuo et al. utilized the corrosion resistance and durability of geopolymers to modify the geopolymer coating of fly ash-slag-based steel with an epoxy emulsion (EP) [7]. Compared with steel bars, the 5% EP-modified geopolymer coating exhibited superior performance. Arokiasamy et al. used silica fume as a partial substitute for kaolinite to create a sustainable geopolymer adsorbent for removing copper ions from synthetic copper solutions, and their research results proved that this polymer could achieve the desired removal effect [8]. Geopolymer gels are expected to have broad application prospects in the future. Therefore, geopolymers are an excellent green cementitious material that keeps pace with the times and can be used to replace cement.

Concrete materials used in hydraulic engineering are often exposed to aggressive environmental conditions. Improving the durability of concrete materials requires dedicated measures. Nanomaterials have been widely utilized across various fields over the past few decades [9]. Civil engineering is one of these fields, where civil engineers are employed to prepare building materials with superior performance. The main nanomaterials used for modifying concrete include nano-SiO_2_ (NS), nano-alumina (NA), and nano-halloysite (Al_2_Si_2_O_5_(OH)_4_), etc. [10]. NS can significantly optimize the microstructure of polymer concrete, which is achieved through multiple mechanisms, including physical filling, chemical activity, and nucleation effects. As a result, the concrete exhibits enhanced mechanical performance, durability, and long-term stability. Due to the unique physical and chemical properties of nanomaterials, an increasing number of scholars are researching their application in GPC. Xu et al. introduced NS into fly ash and coal gangue-based geopolymer concrete (FACGGC), and the results showed that the addition of an appropriate amount of NS markedly improved its performance [11]. NS with smaller particle sizes exhibits a stronger filling effect, whereas NS with larger particle sizes provides better resistance to sulfate erosion. Fried et al. found that 0.1 wt% graphene oxide (GO) improved the mechanical performance, durability, and microstructure of GPC derived from bauxite tailings [12]. Geopolymers exhibit physical and mechanical properties comparable to those of silicate cement. Therefore, it is feasible and effective to add NS to enhance the properties of geopolymers.

Concrete materials used in hydraulic engineering must not only have excellent mechanical properties and high durability, but also good toughness. To enhance the low toughness of GPC, fiber materials can be added to the concrete matrix. Related research results have shown that the toughness of concrete can be enhanced through the incorporation of fibers [13]. Polyvinyl alcohol (PVA) fibers are characterized by high strength, high modulus, and good water dispersion. The addition of PVA fibers can improve the deformation capacity and toughness of concrete [14]. PVA fibers improve crack resistance and performance in concrete at high and low temperatures. Therefore, PVA fibers are ideal for improving the durability of structures in extreme climates. However, PVA fibers still have issues with their ability to improve substrate strength and dispersibility. Steel fibers have excellent reinforcement and toughening effects, which make them ideal for use in high-performance concrete. However, the influence of steel fibers on improving the compressive strength (CS) of the substrate remains controversial [15]. Moreover, steel fibers not only increase the self-weight of concrete because of their high density but also raise the overall cost due to their relatively high price.

Compared to single fibers, the incorporation of a fiber mix in concrete enhances material properties. The enhanced properties encompass energy absorption capacity, ductility, toughness, and durability [16]. A positive hybrid effect can be achieved in cementitious composites by combining high-modulus steel fibers with low-modulus synthetic fibers. Compared with other synthetic fibers, PVA fibers offer a better balance between cost and performance and show particular advantages when combined with steel fibers. The synergistic effect creates a multiscale crack-bridging effect. Steel fibers mainly restrain the development of larger cracks. Meanwhile, PVA fibers help restrain the formation and growth of microcracks [17]. However, if excessive amounts of fibers are added, these fibers are difficult to disperse evenly and will form clumps, which are more prone to cracking. Excessive fiber content can lead to many adverse effects, such as decreased tensile strength, CS, and toughness.

NS particles can densify the matrix by filling microscopic voids and improving the fiber–matrix interface, which reduces the increase in porosity caused by excessive fibers and improves density. Many scholars have studied the synergistic effects of mixed fibers and NS. Zhang et al. studied the workability of self-compacting GPC modified with NS or a steel–PVA hybrid fiber [18]. For self-compacting GPC containing both NS and hybrid fibers, the slump spread, flow rate, and sieve index decreased as the content of NS or fibers increased. Zhang et al. also observed that nano-SiO_2_ and hybrid fiber (NHF) reinforced geopolymer concrete (NHF-GPC) with 1.5% NS, 0.6% PVA fibers, and 1.5% steel fibers exhibited optimal abrasion resistance (AR) [19]. Compared to GPC containing 1.5% NS, the abrasion rate of NHF-GPC decreased by 31.0%, the abrasion strength increased by 43.6%, and the abrasion depth decreased by 43.0%. The CS and splitting tensile strength of NHF-GPC are linearly related to its AR. Zhao et al. investigated the mechanical performance of hybrid fiber and NS reinforced concrete (HFNRC) during exposure to elevated temperatures [20]. The test temperatures covered a range from 200 °C to 800 °C. Over this temperature interval, the compressive and flexural strengths exhibited a decrease–increase–decrease trend. In contrast, a continuous decline was exhibited by the tear tensile strength. At 800 °C, reductions of 63%, 82%, and 76% were observed in the compressive, splitting tensile, and flexural strengths, respectively.

The abrasion resistance of geopolymer gel concrete is primarily governed by the microstructure of its geopolymer gel matrix, which is mainly composed of N-A-S-H or C-A-S-H phases [21]. A dense and well-polymerized gel enhances surface hardness and cohesion, thereby reducing material loss under scouring action. Nano-SiO_2_ promotes gel densification through physical filling and nucleation effects, while hybrid fibers improve the pore structure of the concrete and inhibit crack propagation. Therefore, under the synergistic action of steel fibers, NS, and PVA fibers, the performance of the geopolymer will be greatly improved. Research focusing on determining the most effective proportions of these three components holds great significance for the construction and implementation of water conservancy projects.

Hydraulic concrete structures, due to their long-term contact with water, are inevitably subjected to abrasion from water. Long-term abrasion will not only jeopardize the safety of the hydraulic concrete structures but also increase the cost of structure maintenance. Improving the AR of construction materials is essential for enhancing the service performance and prolonging the service life of hydraulic concrete structures. The optimal proportion of the polymer components is usually achieved through experimental methods. Such an approach consumes considerable manpower and resources and is further limited by a low efficiency and a long testing period. In recent years, modern artificial intelligence has developed rapidly. Data-driven methods, especially machine learning, have been widely adopted. In the study of AR, the proportion of added hybrid fibers and NS significantly influences GPC properties. This relationship is complex and nonlinear. Backpropagation neural networks (BPNN) are particularly effective for modeling this type of relationship. Zhou et al. proposed a new Proportional–Integral–Derivative (PID) control algorithm using edge intelligence, which integrates a BPNN to enhance robustness and adaptability [22]. Simulation experiments show that the PID design based on the BPNN is significantly superior to traditional methods, with the acceleration response time from 0 to 1 m/s improving from 0.25 s to only 0.065 s.

The traditional BPNN is prone to instability and poor generalization capabilities. And the traditional BPNN often produces inconsistent results after multiple training runs and are prone to collapse [23]. Combining the BPNN with optimization algorithms is a common method for improving BPNN. Wang et al. introduced a new prediction model that uses a BPNN optimized by the sparrow search algorithm (SSA) to predict the thermal performance of rooftop greening in subtropical regions [24]. By optimizing the initial weights and thresholds, the SSA-BPNN model achieves better convergence and generalization than the conventional BPNN. Qiu et al. optimized the initial weights and biases of the BPNN using the particle swarm optimization (PSO) algorithm [25]. According to the results, the model integrating the PSO-BPNN and the collaborative filtering algorithm showed the best predictive performance, with a mean squared error (MSE) of 0.019 and a mean absolute error (*MAE*) of 0.037.

Common algorithms for optimizing the BPNN include the Drosophila Optimization Algorithm, the PSO Algorithm, and the Genetic Algorithm. The Grey Wolf Optimizer (GWO) algorithm performs optimization by mimicking the cooperative hunting behavior of gray wolves. The GWO algorithm has a straightforward structure, requires tuning of only a limited number of parameters, and can be readily implemented. Adaptive convergence mechanisms and feedback strategies help maintain a balance between local exploitation and global exploration, resulting in strong performance in solution accuracy and convergence speed. Therefore, in this study, the GWO algorithm is employed to optimize the BPNN for predicting the AR of NHF-GPC, thereby providing guidance for further mix design optimization.

This study has three main objectives. The first objective is to experimentally investigate the AR of NHF-GPC. Both the circular ring test method and the underwater steel ball test method are employed. The evaluation indicators include the abrasion resistance strength, the abrasion rate, and the average abrasion depth. The second objective is to establish a Grey Wolf Optimizer-optimized backpropagation neural network (GWO-BPNN) model for predicting the AR performance of NHF-GPC under varying dosages of NS, PVA fibers, and steel fibers. The third objective is to evaluate the predictive performance of the proposed GWO-BPNN model against the standard BPNN in terms of accuracy, fitting quality, and generalization capability. The ultimate goal is to provide a reliable theoretical basis for the mix design optimization of NHF-GPC in hydraulic engineering applications.

## 2. Results and Discussion

In this study, the AR of NHF-GPC was predicted using the GWO-BPNN and the unoptimized BPNN, respectively. For model development, 70% of the samples were allocated to training, whereas the other 30% were used for testing. Instead of random selection, a stratified sampling strategy was employed to ensure that the testing set covers the full range of mixture proportions. Specifically, five representative groups were selected as the testing set based on their key variables: the control group (C), groups with varying nano-SiO_2_ content (SPN2.0), varying steel fiber content (NPS0.5), and varying PVA fiber content (NSP0.2 and NSP0.4).

Normalization methods are frequently used in deep learning because they help regulate the distribution of inputs within neural networks, thereby improving training stability and accelerating convergence [26]. In this study, linear normalization was used to normalize the data as shown in Equation (1).

(1) $$X_{\mathrm{norm}}=\frac{X-X_{min}}{X_{max}-X_{min}}$$

where $X_{min}$ is the minimum value; $X_{max}$ is the maximum value; X is the sample value; $X_{\mathrm{norm}}$ is the normalized value.

Using Equation (1), the data can be scaled to the [0, 1] or [−1, 1] interval. The minimum value $X_{min}$ and the maximum value $X_{max}$ of the data are first calculated. Each sample X is then scaled according to the formula [27]. The shape of the distribution of the original data is preserved. In this study, this method was used to normalize the data to the range [0, 1].

The abrasion rate of NHF-GPC was determined based on the mass loss of specimens after abrasion testing. For the circular ring test method, the abrasion rate was calculated as the cumulative mass loss percentage after four 30 min cycles. For the underwater steel ball test method, the abrasion rate was obtained from the mass loss of cylindrical specimens after 72 h of rotation at 1200 r/min. For each mix proportion, the average value of three specimens was taken as the test result.

**Figure 1:**
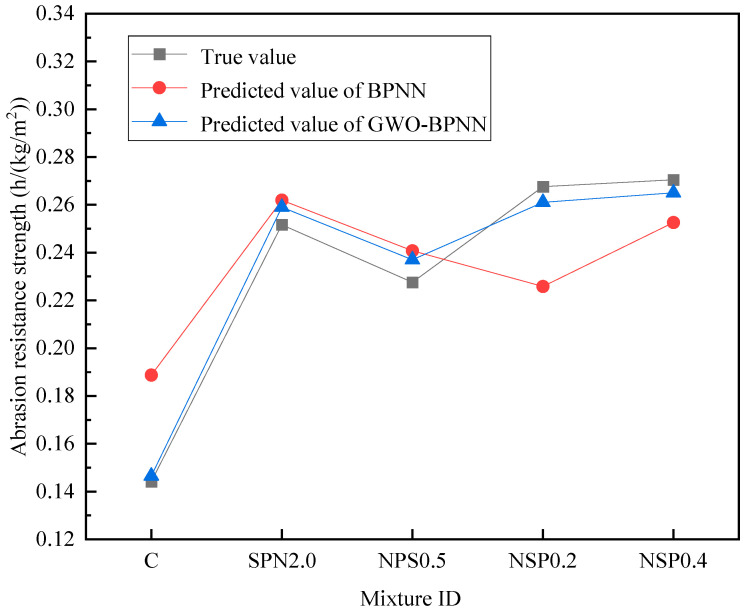
Predicted ARS of NHF-GPC for the circular ring test method.

**Figure 2:**
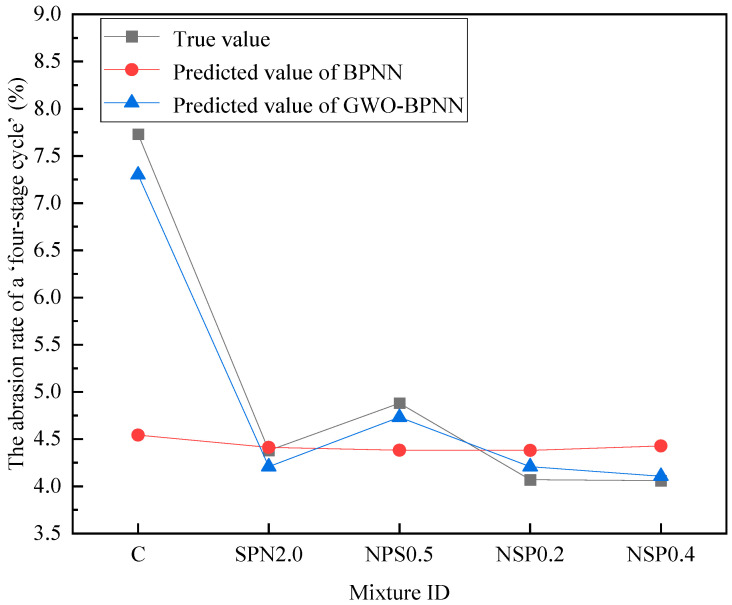
Predicted abrasion rate of NHF-GPC for the circular ring test method.

Figure 1 and Figure 2 represent the true values of the circular ring test method for the SPN2.0, NPS0.5, C, NSP0.2, NSP0.4, as well as the predicted values of the GWO-BPNN, and the predicted values of the BPNN in the same graph. Closer agreement with the real values is observed for the GWO-BPNN-predicted values, in terms of both the abrasion resistance strength (ARS) and the abrasion rate of the “four-stage cycle”. Superior fitting performance is demonstrated by the GWO-BP model. Compared with the control group, the ARS of GPC is enhanced by incorporating NS, PVA fibers, and steel fibers. Concurrently, the abrasion rate is reduced during a “four-stage cycle”.

As shown in Figure 1, the content of NS is held constant. The content of PVA fibers is reduced from 0.6% to 0.2%, while the content of steel fibers is increased from 0.5% to 1.5%. The AR of NHF-GPC is generally increased by the adjustment in fiber composition. The content of NS and steel fibers is held constant. Raising the PVA fiber content from 0.2% to 0.4% leads to a modest increase in the ARS of NHF-GPC. In the abrasion group NSP0.2, the greatest deviation from the true values is observed in the BPNN predictions. A decrease of 15% relative to the true values is recorded for the BPNN. In contrast, a decrease of only 2% is recorded for the GWO-BPNN. From NSP0.2 to NSP0.4, an increase of 1% is observed in the true values. In response, the predicted values of the GWO-BPNN are maintained at nearly identical levels. In contrast, an increase of 12% is recorded for the predicted values of the BPNN. Consequently, superior fitting performance is demonstrated by the GWO-BPNN model. However, AR is not significantly enhanced by raising the PVA fiber content. Excessive PVA fiber content may lead to uneven distribution and agglomeration, thereby increasing internal structural defects and reducing the AR of the concrete [28].

As shown in Figure 2, the control group C is analyzed. The predicted values of the GWO-BPNN are measured at 6% below the true values. In contrast, the predicted values of the BPNN are measured at 41% below the true values. The abrasion groups SPN2.0, NPS0.5, NSP0.2, and NSP0.4 are considered. In these groups, the predicted values of the GWO-BPNN are observed to be very close to the true values. The prediction errors are contained within 4%, which demonstrates high predictive accuracy. At the same time, a significant discrepancy is observed between the BPNN prediction results and the true values. As shown in Figure 2, a very flat trend is exhibited by the BPNN prediction line, whereas the true values follow a markedly different trend. The discrepancy indicates poor-fitting performance. Improperly set initial weights and thresholds in the BPNN can result in convergence to a local optimum, failure to converge, or abnormally slow training [29].

**Figure 3:**
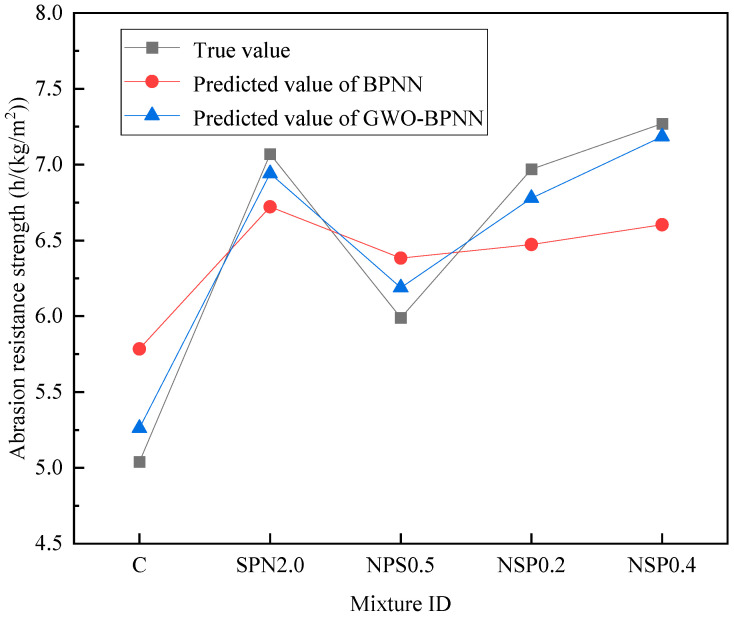
Predicted ARS of NHF-GPC for the underwater steel ball test method.

**Figure 4:**
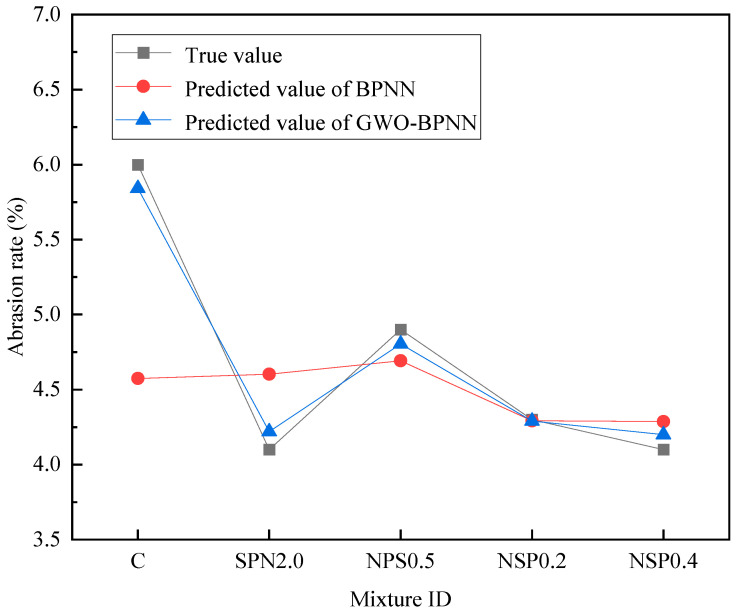
Predicted abrasion rate of NHF-GPC for the underwater steel ball test method.

**Figure 5:**
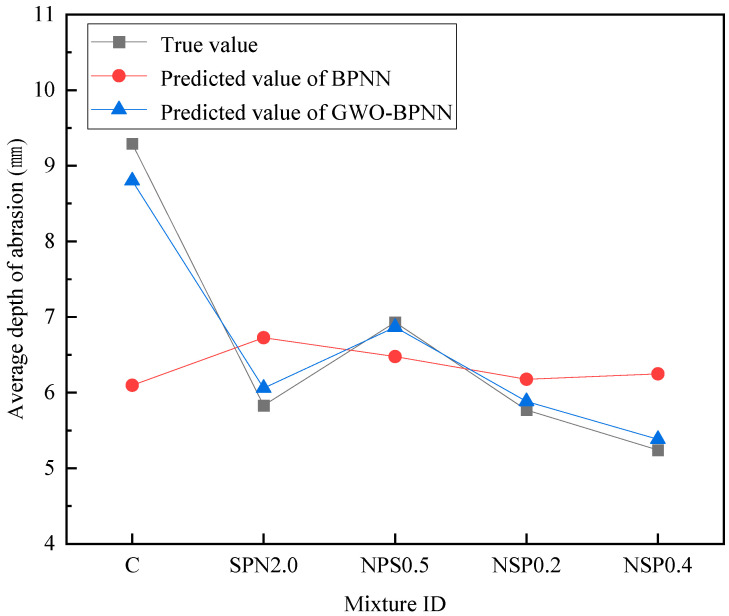
Predicted AAD of NHF-GPC for the underwater steel ball test method.

Based on the underwater steel ball test method, the true values of the groups SPN2.0, NPS0.5, C, NSP0.2, and NSP0.4 are plotted on a single graph. The predicted values from both the GWO-BPNN and the BPNN are also included in this combined plot. In contrast to the circular ring test method, the results for the underwater steel ball test method are presented across three separate figures. The ARS is shown in Figure 3, the abrasion rate in Figure 4, and the average abrasion depth (AAD) in Figure 5.

As shown in Figure 3, the true value shows a significant drop of 15% from SPN2.0 to NPS0.5. A decrease of 11% is recorded for the GWO-BPNN-predicted values. A decrease of 5% is recorded for the BPNN-predicted values. In this case, the NS content decreases from 2% to 1.5%, while the PVA fiber content remains constant and the steel fiber content decreases from 1.5% to 0.5%. When NS and steel fibers are incorporated simultaneously into the concrete, a synergistic effect is produced, which enhances the basic mechanical properties, toughness, and durability of the concrete [30]. Therefore, when the contents of both materials are reduced simultaneously, the synergistic effect is weakened. A significant decrease in AR is then observed. In Figure 3, the predicted values of the GWO-BPNN and the BPNN are on the same side relative to the true values. However, overall, GWO-BPNN has a better fitting quality.

As shown in Figure 4, the predicted values of the GWO-BPNN and the BPNN for the three groups NPS0.5, NSP0.2, and NSP0.4 are very close. In the C and SPN2.0 groups, the predicted results of the GWO-BPNN show better agreement with the actual values. In the control group C, the predicted values of the BPNN are 24% lower than the true values, while the GWO-BPNN-predicted values were 3% lower than the true values. This is similar to the prediction performance of the abrasion rate for the circular ring test method. In Figure 5, the fitting quality of the GWO-BPNN is superior to that of the BPNN.

As shown in Figure 3, Figure 4 and Figure 5, an improvement is observed for both AR test methods. Relative to the control group, the ARS is significantly enhanced in all other groups. Concurrently, the abrasion rate is significantly reduced. Based on the underwater steel ball test method, the AAD is also significantly decreased. The AR of NHF-GPC is significantly improved by the combined action of NS and steel–PVA hybrid fibers. Furthermore, in the prediction of various performance metrics, the GWO-BPNN model demonstrates superior fitting accuracy compared to the BPNN model.

Root mean square error (*RMSE*) [31], *MAE* [32], and mean absolute percentage error (*MAPE*) [33] are adopted to measure the model prediction error and to compare the GWO-BP and BP models. These metrics are calculated by Equations (2)–(4).

(2) $$RMSE=\sqrt{\frac{1}{n}\sum_{i=1}^{n}{\left(y_{i}-{\hat{y}}_{i}\right)}^{2}}$$

(3) $$MAE=\frac{1}{n}\sum_{i=1}^{n}\left|y_{i}-{\hat{y}}_{i}\right|$$

(4) $$MAPE=\frac{100\%}{n}\sum_{i=1}^{n}\left|\frac{y_{i}-{\hat{y}}_{i}}{y_{i}}\right|$$

where n is the total number of samples, while $y_{i}$ and ${\hat{y}}_{i}$ are the observed and predicted values of the *i*-th sample, respectively.

Table 1, Table 2, Table 3, Table 4 and Table 5 demonstrate the prediction error analyses of the AR performance metrics of NHF-GPC under the circular ring test method and the underwater steel ball test method by the GWO-BPNN and BPNN models. Analysis of the data shows that the AR prediction error of GWO-BPNN for NHF-GPC is less than that of BPNN. For GWO-BPNN, the *RMSE* value and *MAE* value in the abrasion rate prediction of the circular ring test method are the highest, while the *MAPE* in the ARS prediction of the circular ring test method is the highest. For the underwater steel ball test method, small prediction errors are observed across all parameters. Additionally, a minimal deviation is found between the model predictions and the true values. However, overall, the prediction errors are all within a reasonable range, which demonstrates good prediction accuracy. The research results of Chen et al. indicate that the GWO algorithm has greater advantages in terms of global exploration capability and convergence efficiency [34]. The applicability of the GWO-BPNN for predicting the AR of NHF-GPC is demonstrated. Good fitting effects and high prediction accuracy are achieved. Consequently, practical guidance for related hydraulic engineering applications is provided.

## 3. Conclusions

(1) The addition of NS and steelPVA hybrid fibers substantially enhanced the AR of GPC. Based on the circular ring test method, the ARS of the control group was 0.14 h/(kg/m^2^), while the optimal mix (SPN1.5) reached 0.29 h/(kg/m^2^), representing a 107.1% increase. The abrasion rate decreased from 7.73% to 3.78%, a reduction of 51.1%. Based on the underwater steel ball test method, the ARS increased from 5.04 h/(kg/m^2^) to 7.38 h/(kg/m^2^), an improvement of 46.4%, while the abrasion rate decreased from 6% to 4%. The AAD was reduced from 9.29 mm to 4.86 mm, a decrease of 47.7%. These results demonstrate that NS and steel–PVA hybrid fibers effectively enhance the toughness and durability of GPC.

(2) The GWO-BPNN model demonstrates superior prediction performance for the AR of NHF-GPC compared to the conventional BPNN. For the circular ring test method, the GWO-BPNN achieved *RMSE*, *MAE*, and *MAPE* values of 0.035, 0.025, and 14.2% for the ARS prediction, and 1.123, 0.581, and 8.4% for abrasion rate prediction. In comparison, the BPNN yielded corresponding values of 0.052, 0.031, 19.0% and 1.611, 0.867, 12.7%, respectively. The *RMSE* for abrasion rate prediction was reduced by 30.3%, and *MAPE* was lowered from 12.7% to 8.4%. For the underwater steel ball test method, the GWO-BPNN achieved *RMSE* values of 0.342, 0.358, and 0.404 for the ARS, abrasion rate, and AAD predictions, with *MAPE* values of 5.1%, 5.7%, and 5.5%, respectively. In contrast, the BPNN yielded *RMSE* values of 1.066, 0.750, and 1.291, with *MAPE* values of 14.6%, 11.3%, and 15.3%. Across all five prediction tasks, the GWO-BPNN reduced the *RMSE* by 30.3–68.7% and the *MAPE* by 25.3–65.1% compared to the BPNN, indicating significantly higher prediction accuracy, better fitting performance, and stronger generalization capability.

(3) The GWO-BPNN model established in this study provides an efficient and accurate method for predicting the AR of NHF-GPC. The model takes the NS dosage (0–2.0%), the PVA fiber dosage (0–0.8%), the steel fiber dosage (0–2.0%), and the fly ash content as input variables, and outputs the ARS, the abrasion rate, and the AAD under different test methods. With *MAPE* values ranging from 5.1% to 14.2% across all prediction tasks, the model can rapidly predict the AR of NHF-GPC at various mix proportions without conducting time-consuming and labor-intensive experiments. This offers reliable theoretical guidance and technical support for the mix design optimization and performance prediction of NHF-GPC in hydraulic engineering applications, ultimately contributing to the development of more durable and sustainable hydraulic concrete structures. In future studies, standard deviation estimates will be incorporated to further characterize the variability of experimental measurements, and the proposed GWO-BPNN model will be extended to account for prediction uncertainty, thereby strengthening its applicability in practical engineering.

## 4. Materials and Methods

### 4.1. Experimental Program

In this study, the key parameters of the mix design for NHF-GPC include the fly ash and slag dosages, aggregates, alkali activators, and water content. A prediction model is developed using a GWO-optimized BPNN. The input variables for the model contain the dosages of steel fibers, PVA fibers, NS, and fly ash. For the circular ring test method of NHF-GPC, the study uses the ARS and a “four-stage cycle” abrasion rate as the output values. For the underwater steel ball test method, the output values are the ARS, the abrasion rate, and the AAD.

#### 4.1.1. Raw Materials

NHF-GPC was prepared from fly ash, slag, water glass, river sand, natural gravel, NS, steel–PVA hybrid fibers, and NAOH. NS and steel–PVA hybrid fibers were used as the modifying materials, and the basic properties of NS, PVA fibers, and steel fibers are presented in Table 6 and Table 7. Fly ash has a spherical microstructure, and slag has a tetragonal structure. The main chemical properties of slag and fly ash are shown in Table 8 and Table 9. An alkaline activator with a silicate modulus of 1.3 was prepared by blending water glass with sodium hydroxide at a predetermined ratio. The coarse aggregate consisted of natural gravel, and the fine aggregate consisted of natural river sand. The river sand used in the experiments was coarse sand with a fineness modulus of 3.0. The particle grading of the crushed stone used was a continuous grading with a nominal grain size of 5–20 mm, and the distribution of grain size was in accordance with the specification.

#### 4.1.2. Mix Proportions of NHF-GPC

The study examined how the incorporation ratio of NS and steel–PVA hybrid fibers affects the AR of NHF-GPC. In this study, the amount of reinforcement was taken as a core variable in the formulation of the proportioning scheme. In this experiment, the control variable method was employed to determine the key parameters, which included the ratios of fly ash to slag, water to binder, and aggregate to binder, as well as the sand ratio in the system. Only the dosages of steel fibers, NS, and PVA fibers were varied.

Following continuous experimental adjustments, the final mix proportions were determined. The fly ash-to-slag mass ratio was fixed at 4:1. A water-to-binder ratio of 0.45 was adopted, with a sand ratio of 40% and a bone-cement ratio of 3.0. The original modulus of the water glass was 3.2. This modulus was adjusted using 99.0% pure, white flake NaOH. The final modulus of the water glass was adjusted at 1.3. Fly ash and slag were replaced by an equal mass of NS at dosage levels of 0%, 0.5%, 1.0%, 1.5%, and 2.0%, while all other proportioning parameters were fixed. The mix proportions of NHF-GPC are detailed in Table 10.

#### 4.1.3. AR Test and Results

In this study, two forms of AR tests were performed on NHF-GPC with varying dosages of NS and hybrid fibers. The first was the circular ring test method, which simulates suspended mass abrasion. The second was the underwater steel ball test method, which simulates nudged mass abrasion. Different parameters of AR are employed for these two experiments. The prediction model employed is a GWO-BPNN with an input layer, a hidden layer, and an output layer. In the circular ring test of NHF-GPC, the ARS and the abrasion rate over a four-stage cycle were adopted as the evaluation parameters. This selection was made because the abrasion rates obtained from four repeated trials on the same NHF-GPC proportion showed some variation. However, a similar variation pattern was maintained across different mix proportions. In order to assess the AR of NHF-GPC through the underwater steel ball test method, the ARS, the abrasion rate, and the average depth of abrasion were taken as the assessment parameters for the AR performance.

Based on the circular ring test method, each set of ratios underwent four abrasion tests. The residual mass of the test piece was weighed at half-hourly intervals during the conduct of the test and recorded four times in succession. Table 11 presents the experimental results.

The circular ring test method is used to simulate the suspended sediment erosion effect. Circular ring specimens are employed, along with diamond sand and water. Each test abrasion session lasts for 30 min, and a total of 4 sessions are conducted. The total cumulative quality loss over the 4 sessions is recorded as the quality loss of the specimen. The ARS of concrete based on the circular ring test method can be calculated using Equations (5) and (6).

(5) $$R_{a}=\frac{TA}{M_{T}}$$

(6) A=πDH

where $R_{a}$ is the ARS of concrete (h/(kg/m^2^)), T is the abrasion duration (h), D and H are the inner diameter and height of the circular specimen (m), $M_{T}$ is the abrasion-induced mass loss of the specimen (kg).

Based on the underwater steel ball test method, the mass of the specimens before and after the abrasion test was recorded, and the abrasion rate of the NHF-GPC was calculated using Equation (7). The average value of three specimens for each set of ratios was taken as the test result. The experimental results are presented in Table 12.

The underwater steel ball test method is used to simulate the sediment transport erosion effect. A cylindrical sample is placed in an abrasion tester filled with steel balls and water, and it rotates at 1200 r/min for 72 h. The mass loss of the sample before and after the abrasion process is recorded. The abrasion rate of concrete can be calculated using Equation (7).

(7) $$L_{a}=\frac{M_{0}-M_{t}}{M_{0}}\times 100\%$$

where $L_{a}$ is the concrete abrasion rate (%), $M_{0}$ is the initial specimen mass (kg), and $M_{t}$ is the specimen mass after punching and abrasion (kg).

The ARS of concrete can be calculated using Equation (8).

(8) $$R_{a}=\frac{TA}{M_{T}}$$

where $R_{a}$ is the concrete ARS (h/(kg/m^2^)), A is the area of the specimen exposed to abrasion (m^2^).

### 4.2. Model Establishment

In this study, fly ash, slag, sodium silicate, NaOH, river sand, and natural gravel were utilized as raw materials. A GWO-BPNN was employed as the analytical method. The combined effect of steel–PVA hybrid fibers and the NS admixture on the AR of NHF-GPC was investigated. A BPNN is composed of three layers: the input layer, the hidden layer, and the output layer. As described in Part II, four material parameters influencing the AR of NHF-GPC were identified, namely the dosages of NS, PVA fiber, steel fiber, and fly ash. These parameters are used as the input layer, which consists of four neurons. In the AR test method of NHF-GPC, based on the circular ring test method, the ARS and the abrasion rate of a “four-stage cycle” are used as the output values, which means that the output layer includes two neurons. In the experiments based on the underwater steel ball test method, the ARS, the abrasion rate, and the AAD were adopted as the output variables, corresponding to an output layer with three neurons.

#### 4.2.1. BPNN Model

**Figure 6:**
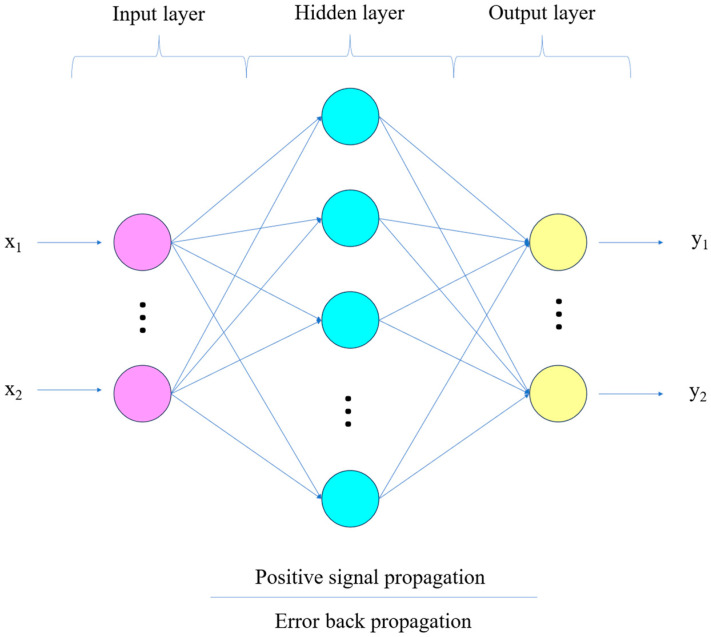
Structure of the BPNN.

Artificial neural networks (ANNs) are data-driven models designed to mimic certain functions of the human brain. Through adaptive learning, ANNs can capture and represent complex relationships in large datasets [35]. ANN models can be classified into hierarchical and interconnected types according to the structural features of biological neural networks. Hierarchical neural network models are typically trained using the BP algorithm [36]. The BP neural network is a multilayer feedforward network trained through the error backpropagation algorithm. BPNNs are widely applied in practical ANN tasks, including function approximation, pattern recognition and classification, and data compression [37]. The BPNN consists of an input layer, a hidden layer, and an output layer. During forward propagation, data flow from the input layer through the hidden layer to the output layer in sequence. The sequential processing activates the nodes at each stage, ultimately producing a predicted result. A minor discrepancy may occur between the predicted output and the actual value due to various factors. An error function is selected, and the gradients of w and b are adjusted using the gradient descent method to obtain the corrected values. Finally, the trained model is validated, and its predictive capability is assessed in terms of accuracy. In this study, since NS replaces fly ash and slag an equal mass, the mass ratio of fly ash to slag is kept constant. Therefore, the steel fiber dosage, PVA fiber dosage, NS dosage, and fly ash dosage are taken as the inputs, which means the input layer contains four neurons. The number of neurons in the output layer is set to two for the circular ring test method and three for the underwater steel ball test method. A common empirical practice is used to initialize the hidden layer. A common practice is to set the number of neurons in the hidden layer to approximately 1 to 2 times the number in the input layer. In this study, eight neurons are set in the hidden layer. The model structure of the BPNN is shown in Figure 6.

The prediction of a certain performance using the BPNN mainly consists of several steps: forward propagation, loss computation, backpropagation, and weight adjustments. The situation is as follows:

##### Forward Propagation

The data is passed from the input layer through sequential processing, which includes weight calculation and activation function operations. And the prediction result is finally obtained at the output layer. Forward propagation is the fundamental process of the BPNN, which provides the necessary inputs for subsequent loss calculation and parameter optimization.

Activation functions are an important component of neuron operations in BPNN. Activation functions introduce nonlinear features into neural networks, thereby enabling neural networks to perform nonlinear transformations. The key purpose of activation functions is to transform neuron inputs in specific ways and map the inputs to outputs. Through this process, neural networks can handle complex real-world patterns [38]. Zhang et al. employed the ReLU function in their model and used the BPNN to predict the rheological properties of GM with NS and PVA fiber reinforcement [39]. When Amiri used ANNs to predict the mechanical and durability characteristics of concrete, all neurons used the tangent sigmoid transfer function [40], while Yang et al. used the tanh function as the activation function in the combined wool spinning process parameter inversion model [41]. The ReLU function, tanh function, and sigmoid function can be presented in Equations (9)–(11).

(9) $$f(x)=\left\{\begin{array}{lll} 0 & for\;x<0 & \\ x & for\;x\geq 0 & \end{array}\right.$$

(10) $$f(x)=\frac{1}{1+e^{-x}}$$

(11) $$f(x)=\tanh(x)=\frac{\left(e^{x}-e^{-x}\right)}{\left(e^{x}+e^{-x}\right)}=2sigmoid(2x)-1$$

Zhang et al. pointed out that the ReLU function is more popular in multilayer neural networks [39]. The ReLU function has a constant gradient of 1 for positive inputs, which avoids the gradient vanishing problem of traditional saturation activation functions and accelerates the convergence speed of backpropagation. The ReLU function demonstrates clear advantages in stability when dealing with complex problems and is also better suited for training deep neural networks [42]. Accordingly, the ReLU function was adopted as the hidden-layer activation function in this study. The output layer employed the purelin linear activation function. Hong et al. believe that this function retains the numerical scaling of the preceding layers. [43]. The purelin activation function can be shown in Equation (12).

(12) f(z)=z

The data is forward-propagated with weighted inputs and activated outputs in sequence. The weighted input of the hidden layer is presented in Equation (13), and the activation output is presented in Equation (14).

(13) $$z^{\left(1\right)}=W^{\left(1\right)}x+b^{\left(1\right)}$$

(14) $$a^{\left(1\right)}=ReLU\left(z^{\left(1\right)}\right)=\max\left(0,z^{\left(1\right)}\right)$$

The weighted inputs to the output layer are shown in Equation (15), and the predicted outputs are shown in Equation (16).

(15) $$z^{\left(2\right)}=W^{\left(2\right)}a^{\left(1\right)}+b^{\left(2\right)}$$

(16) $$a^{\left(2\right)}=z^{\left(2\right)}$$

where $W^{\left(1\right)}$ and $b^{\left(1\right)}$ are the weight matrix and bias vector associated with the hidden layer, $W^{\left(2\right)}$ and $b^{\left(2\right)}$ are the weight matrix and bias vector associated with the output layer, x is the input vector, $z^{\left(1\right)}$ and $a^{\left(1\right)}$ are the weighted input and the activated output associated with the hidden layer, $z^{\left(2\right)}$ and $a^{\left(2\right)}$ are the weighted inputs and the predicted values associated with the output layer.

##### Loss Computation

Predicted outputs are generated through forward propagation. A discrepancy is observed between these predictions and the ground truth values. The discrepancy is then utilized as the basis for gradient-based optimization in the backpropagation algorithm. Common loss functions are the MSE [44], the *MAE* [45], the Huber Loss [46], and so on. In this study, the MSE is used as the global error function, as shown in Equation (17).

(17) $$L=\frac{1}{2}\sum_{i=1}^{k}{\left(y_{i}-a_{i}^{\left(2\right)}\right)}^{2}$$

where L is the loss function, $y_{i}$ is the *i*-th true value, and $a_{i}^{\left(2\right)}$ is the *i*-th output layer prediction.

##### Back Propagation

The error is first computed according to step (2). Then, the error is reduced along the direction of the parameters w and b using the gradient descent method. More specifically, the error term is transmitted from the output layer back to the hidden layer. The error term of the last output layer can be computed as Equation (18).

(18) $$\delta^{\left(2\right)}=\frac{\partial L}{\partial z^{\left(2\right)}}=a^{\left(2\right)}-y$$

The derivation process of Equation (18) is shown in Equation (19).

(19) $$\frac{\partial L}{\partial z^{\left(2\right)}}=\frac{\partial L}{\partial a^{\left(2\right)}}\cdot \frac{\partial a^{\left(2\right)}}{\partial z^{\left(2\right)}}=\left(a^{\left(2\right)}-y\right)⊙1=a^{\left(2\right)}-y$$

The error term $\delta^{\left(1\right)}$ of the hidden layer can be calculated using Equation (20).

(20) $$\delta^{\left(1\right)}=\left(W^{\left(2\right)T}\delta^{\left(2\right)}\right)⊙ReLU^{\prime }\left(z^{\left(1\right)}\right)$$

The derivative of the ReLU function is shown in Equation (21).

(21) $$ReLU^{\prime }\left(z_{i}^{\left(1\right)}\right)=\{\begin{array}{ll} 1 & if\;z_{i}^{\left(1\right)}>0\\ 0 & otherwise \end{array}$$

where $\delta^{\left(2\right)}$ is the error term of the output layer, $\delta^{\left(1\right)}$ is the error term of the hidden layer, ⊙ is element-by-element multiplication.

##### Weighting Adjustments

The weights and biases are updated using gradient descent to enhance the performance of the model. The weights and bias gradients of the output layer are shown in Equations (22) and (23).

(22) $$\frac{\partial L}{\partial W^{\left(2\right)}}=\delta^{\left(2\right)}a^{(1)T}$$

(23) $$\frac{\partial L}{\partial b^{\left(2\right)}}=\delta^{\left(2\right)}$$

The updated formulas are shown in Equations (24) and (25).

(24) $$W^{\left(2\right)}\leftarrow W^{\left(2\right)}-\eta \cdot \frac{\partial L}{\partial W^{\left(2\right)}}$$

(25) $$b^{\left(2\right)}\leftarrow b^{\left(2\right)}-\eta \cdot \frac{\partial L}{\partial b^{\left(2\right)}}$$

The weights and bias gradients of the hidden layer are shown in Equations (26) and (27).

(26) $$\frac{\partial L}{\partial W^{\left(1\right)}}=\delta^{\left(1\right)}x^{T}$$

(27) $$\frac{\partial L}{\partial b^{\left(1\right)}}=\delta^{\left(1\right)}$$

The updated formulas are shown in Equations (28) and (29).

(28) $$W^{\left(1\right)}\leftarrow W^{\left(1\right)}-\eta \cdot \frac{\partial L}{\partial W^{\left(1\right)}}$$

(29) $$b^{\left(1\right)}\leftarrow b^{\left(1\right)}-\eta \cdot \frac{\partial L}{\partial b^{\left(1\right)}}$$

where η is the learning rate.

**Figure 7:**
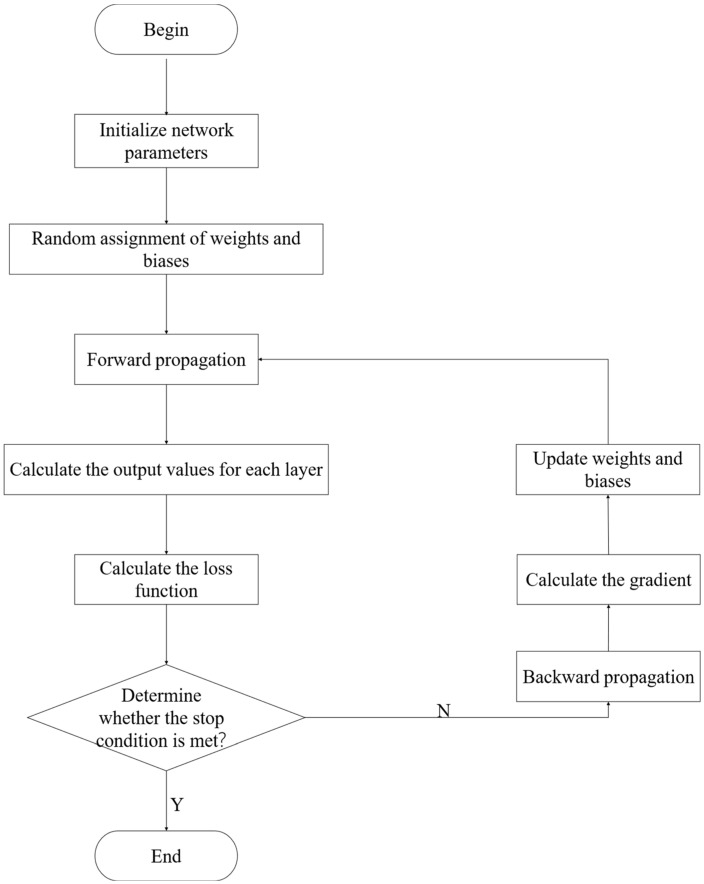
Flowchart of BPNN.

The above steps are computed iteratively. The BPNN is effectively trained by this process. The flowchart of the BPNN is presented in Figure 7. During the training process of a BP neural network, the network structure is first determined and the hyperparameters are set. Then, random initial values are assigned to the connection weights and biases of the network. Subsequently, forward propagation is executed, and the output values of the network for each training sample are calculated layer by layer. The loss function is then computed based on the output and the true label. Next, it is determined whether the preset stopping condition is met. If it is, the training ends. If not, the backpropagation stage is entered, and the gradients of the loss function with respect to each parameter are calculated layer by layer using the chain rule. The weights and biases are updated based on the gradients. After the parameter update is completed, the forward propagation step is returned to, and the output values and the loss function are recalculated under the new parameters. The stopping condition is checked again. This cycle is repeated until the stopping condition is met.

#### 4.2.2. Grey Wolf Optimizer Algorithm

The gray wolf is a carnivorous member of the Canidae family. Most gray wolves live in packs with a strict social hierarchy, and they hunt cooperatively by taking on different roles according to their rank, which maximizes their success in capturing prey. A new way of thinking is provided to researchers by the group performance of gray wolves. In the context of the evolving optimization of group intelligence, the GWO algorithm was born. The algorithm is a metaheuristic optimization strategy that mimics the hunting performance of gray wolves [47]. In 2014, Mirjalili et al. presented the GWO algorithm [48]. Águila-León et al. analyzed the introduction of the GWO to enhance the PSO algorithm [49]. In a photovoltaic system, the optimized PSO algorithm was embedded into the Maximum Power Point Tracking (MPPT) controller. The results showed that integrating the GWO-optimized PSO-MPPT algorithm led to improved MPPT controller performance. Göppel et al. introduced a Grey Wolf Optimization-based trajectory adjustment method, where traffic complexity was mitigated by making small lateral changes to the flight path [50]. Significant improvements in key metrics were observed in two case studies. The reductions were 27% in average traffic complexity and 30% in peak complexity load. The specific details of the GWO algorithm are as follows.

The social hierarchy of gray wolves is divided into four levels, α wolves, β wolves, δ wolves, and ω wolves. The α wolves are superior to β and δ wolves [47]. The hierarchy is strictly enforced, with orders being issued from the highest to the lowest levels. Members at each level carry out their duties in an orderly manner, efficiently completing collective hunts.

To describe the social hierarchy mathematically, the GWO algorithm uses the three search agents ranked highest by fitness to represent the leading wolves in the pack. Other individuals cooperate in hunting under the guidance of the three gray wolves [51]. The stages of the GWO algorithm are as follows:

##### Encircling the Prey Stage

The gray wolf encirclement behavior during the hunt can be defined by Equations (30)–(33).

(30) $$\vec{D}=\left|\vec{C}\cdot {\vec{X}}_{P}(t)-\vec{X}(t)\right|$$

(31) $$\vec{X}(t+1)={\vec{X}}_{p}(t)-\vec{A}\cdot \vec{D}$$

(32) $$\vec{A}=2\vec{a}\cdot {\vec{r}}_{1}-\vec{a}$$

(33) $$\vec{C}=2\cdot {\vec{r}}_{2}$$

where Equation (26) is the distance between an individual wolf and the prey, Equation (27) is the updated formula for the wolf location derived from Equation (26), t is the number of iterations completed so far, $\vec{A}$ and $\vec{C}$ are coefficient vectors and ${\vec{X}}_{p}$ is the position vector of the prey, $\vec{X}$ is the position vector of the wolf, $\vec{a}$ is a convergence factor that declines from 2 to 0 as the iterations proceed, The modes of ${\vec{r}}_{1}$ and ${\vec{r}}_{2}$ are random numbers within the interval [0, 1].

##### Hunting Stage

The location of the prey is first determined. The pack is then led by the α wolf. Finally, the prey is surrounded. The three optimal solutions are designated as α, β, and δ. These wolves are positioned nearest to the prey. The location of prey can be determined based on the positions of α, β, and δ. Finally, the prey is gradually approached and captured. The distances between the leading wolves α, β, and δ and the remaining individuals are given by Equation (34).

(34) $$\left\{\begin{array}{l} {\vec{D}}_{\alpha }=|{\vec{C}}_{1}\cdot {\vec{X}}_{\alpha }-\vec{X}|\\ {\vec{D}}_{\beta }=|{\vec{C}}_{2}\cdot {\vec{X}}_{\beta }-\vec{X}|\\ {\vec{D}}_{\delta }=|{\vec{C}}_{3}\cdot {\vec{X}}_{\delta }-\vec{X}| \end{array}\right.$$

where $\vec{X}$ is the current position vector of the gray wolf, the current positions of α, β, and δ are represented by ${\vec{X}}_{\alpha }$, ${\vec{X}}_{\beta }$, and ${\vec{X}}_{\delta }$, respectively. ${\vec{D}}_{\alpha }$, ${\vec{D}}_{\beta }$, and ${\vec{D}}_{\delta }$ are the distances between α, β, and δ and other remaining individuals, respectively, and ${\vec{C}}_{1}$, ${\vec{C}}_{2}$, and ${\vec{C}}_{3}$ are random vectors.

The procedure of updating the position of ω led by α, β, and δ can be calculated using Equation (35). The final position of ω be calculated using Equation (36).

(35) $$\left\{\begin{array}{l} {\vec{X}}_{1}={\vec{X}}_{\alpha }-A_{1}\cdot ({\vec{D}}_{\alpha })\\ {\vec{X}}_{2}={\vec{X}}_{\beta }-A_{2}\cdot ({\vec{D}}_{\beta })\\ {\vec{X}}_{3}={\vec{X}}_{\delta }-A_{3}\cdot ({\vec{D}}_{\delta }) \end{array}\right.$$

(36) $$\vec{X}(t+1)=\frac{{\vec{X}}_{1}+{\vec{X}}_{2}+{\vec{X}}_{3}}{3}$$

#### 4.2.3. GWO-BPNN Model

**Figure 8:**
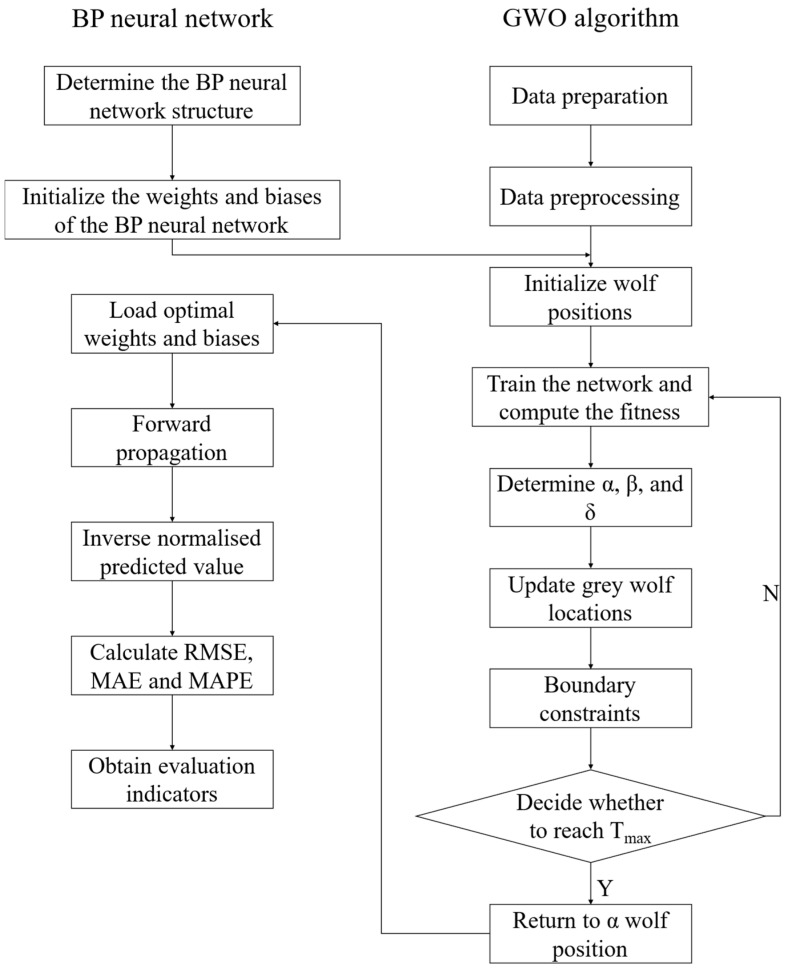
Flowchart of the GWO-BPNN.

The weights in the traditional BPNN are updated using the gradient descent method. Consequently, the network is prone to converging to local optima. Furthermore, the network exhibits slow convergence, low prediction accuracy, and sensitivity to the initial weights and thresholds. To improve these shortcomings, GWO is introduced to improve the BPNN. Gao et al. proposed a GWO-BP hybrid model that combines a GWO-based semi-empirical model with a BPNN-based error prediction model [52]. Yang et al. developed an improved Grey Wolf Optimizer–BPNN approach to predict the remaining useful life (RUL) of vehicle-oriented Proton Exchange Membrane Fuel Cell (PEMFC) systems under actual traffic conditions, with the relative power loss rate (RPLR) adopted as the health indicator [53]. The heart of the GWO-BPNN model lies in optimizing the weight and threshold parameters of the BPNN. First, the required BPNN is constructed. Next, the weights and thresholds of the BPNN are extracted. The GWO algorithm is then employed to optimize these parameters through its global search capability. Finally, the optimized parameters are returned to the BPNN [54]. The flowchart of the GWO-BPNN is presented in Figure 8.

The detailed calculation steps of the GWO-BPNN are as follows.

##### Flattening of Weights and Biases in BPNN

The GWO algorithm handles vector-form data. Therefore, the adjustable parameters of the BPNN must be flattened into a vector. The parameter extraction is conducted as follows.

First, the dimensions of the weight matrix W_in_ between the input and hidden layers are 4 × 8, giving a total of 32 elements. Second, the threshold vector B of the hidden layer has dimensions 8 × 1 = 8. Third, the weight matrix W between the hidden and output layers is sized 8 × 2 for the circular ring test method and 8 × 3 for the underwater steel ball test method, corresponding to 16 and 24 elements, respectively. Fourth, the threshold vector B of the output layer has dimensions of 2 × 1 = 2 for the circular ring test method and 3 × 1 = 3 for the underwater steel ball test method.

The weight matrix and the threshold vector are flattened by splicing them sequentially into a one-dimensional vector. Tis yields a total dimension of 32 + 8 + 16 + 2 = 58 for the circular ring test method experiments and 32 + 8 + 24 + 3 = 67 for the underwater steel ball test method experiments.

To assess the performance of each set of parameters (i.e., the location of each wolf), the fitness function was defined as shown in Equation (37).

(37) Fitness=MSE

The position of the wolf is first decoded into the weights and biases of the BP network. These parameters are then used to perform forward propagation. Forward propagation yields a prediction result. Finally, the MSE is calculated based on this prediction. The fitness function calls the MSE directly, and the optimization objective is to minimize the fitness (i.e., minimize the MSE).

##### GWO Algorithm Global Search

After flattening all the adjustable parameters of the BPNN, the GWO algorithm is used for a global search. The specific procedure is as follows.

First, the wolf pack is first initialized by setting the population size N to 60, the maximum number of iterations T_max_ to 400, and the search space to [−1, 1]. Based on the problem dimension, N random position vectors of gray wolves are generated, each uniformly distributed within the search space. Next, the iterative optimization process begins, where the position vector of each wolf is decoded into the parameters of the BPNN and its fitness value is calculated. According to the fitness in descending order, the top three wolves are identified as α, β, and δ. The position of each gray wolf is then updated using the position update formula, and any components that exceed the search space are truncated to the boundary values. The optimization process stops when either the maximum iteration number is reached or the fitness value no longer improves markedly. Finally, the position vector of α is chosen as the globally optimal parameter combination.

##### BPNN Performs Forward Propagation for Prediction

The optimization of weights and thresholds in the GWO-BPNN is performed using the GWO algorithm, rather than through the gradient descent method employed in conventional BP networks. The weights and biases are first optimized by the GWO algorithm. These optimized parameters are then applied within the BPNN architecture. Finally, forward propagation is executed to obtain the predicted values.

The GWO-BPNN achieves excellent model performance through the aforementioned staged optimization. The GWO algorithm significantly outperforms other intelligent algorithms, such as evolutionary strategies, genetic algorithms, and the PSO algorithms in terms of global optimization [55]. The GWO algorithm effectively escapes local optima and achieves a group search to cover a wider solution space. These capabilities significantly improve the accuracy and precision of the model predictions [56,57,58]. The GWO-BPNN has widespread applications in various practical fields.

## Figures and Tables

**Table 1:** Prediction errors of ARS for the circular ring test method.

NN Models	*RMSE*	*MAE*	*MAPE*
GWO-BPNN	0.035	0.025	14.2%
BPNN	0.052	0.031	19.0%

**Table 2:** Prediction errors of abrasion rate for the circular ring test method.

NN Models	*RMSE*	*MAE*	*MAPE*
GWO-BPNN	1.123	0.581	8.4%
BPNN	1.611	0.867	12.7%

**Table 3:** Prediction errors of ARS for the underwater steel ball test method.

NN Models	*RMSE*	*MAE*	*MAPE*
GWO-BPNN	0.342	0.321	5.1%
BPNN	1.066	0.848	14.6%

**Table 4:** Prediction errors of abrasion rate for the underwater steel ball test method.

NN Models	*RMSE*	*MAE*	*MAPE*
GWO-BPNN	0.358	0.287	5.7%
BPNN	0.750	0.568	11.3%

**Table 5:** Prediction errors of the AAD for the underwater steel ball test method.

NN Models	*RMSE*	*MAE*	*MAPE*
GWO-BPNN	0.404	0.334	5.5%
BPNN	1.291	1.042	15.3%

**Table 6:** Properties of NS.

Specific Surface Area (m^2^/g)	Quantity Contained (%)	Average Particle Size (nm)	pH Value	Apparent Density (g/L)
220	99.5	30	6	55

**Table 7:** Properties of PVA/steel fiber.

Fiber Variety	Diameter (μm)	Standard Length (mm)	Young Modulus (GPa)	Tensile Strength (MPa)
PVA fiber	40	12	41	1560
Steel fiber	210	13	200	2750

**Table 8:** Main chemical compositions of fly ash.

Chemical Compositions	SiO_2_	Al_2_O_3_	Fe_2_O_3_	CaO	MgO	f-CaO	SO_3_	Other
Contents (wt%)	60.98	24.47	6.70	4.90	0.68	0.58	0.52	1.17

**Table 9:** Main chemical compositions of slag.

Chemical Compositions	CaO	SiO_2_	Al_2_O_3_	MgO	SO_3_	TiO_2_	K_2_O	Other
Contents (wt%)	39.25	33.40	15.15	7.67	2.38	0.62	0.39	1.14

**Table 10:** Mixture proportions of the NHF-GPC.

Mix Number	Fly Ash	Slag	Water Glass	NAOH	Water	Fine Aggregate	Coarse Aggregate	NS	PVA Fiber	Steel Fiber
	kg/m^3^							%		
C	420	105	168.6	26.4	125	630	945	0	0	0
SPN0	420	105	168.6	26.4	125	630	945	0	0.6	1.5
SPN0.5	417.9	104.5	168.6	26.4	125	630	945	0.5	0.6	1.5
SPN1.0	415.8	104.0	168.6	26.4	125	630	945	1.0	0.6	1.5
SPN1.5	413.7	103.4	168.6	26.4	125	630	945	1.5	0.6	1.5
SPN2.0	411.6	102.9	168.6	26.4	125	630	945	2.0	0.6	1.5
NPS0	413.7	103.4	168.6	26.4	125	630	945	1.5	0.6	0
NPS0.5	413.7	103.4	168.6	26.4	125	630	945	1.5	0.6	0.5
NPS1.0	413.7	103.4	168.6	26.4	125	630	945	1.5	0.6	1.0
NPS2.0	413.7	103.4	168.6	26.4	125	630	945	1.5	0.6	2.0
NSP0	413.7	103.4	168.6	26.4	125	630	945	1.5	0	1.5
NSP0.2	413.7	103.4	168.6	26.4	125	630	945	1.5	0.2	1.5
NSP0.4	413.7	103.4	168.6	26.4	125	630	945	1.5	0.4	1.5
NSP0.8	413.7	103.4	168.6	26.4	125	630	945	1.5	0.8	1.5

**Table 11:** Results of resistance to abrasion based on the circular ring test method [20].

Mix Number	ARS	Abrasion Rate
	h/(kg/m^2^)	%
C	0.14	7.73
SPN0	0.25	4.4
SPN0.5	0.27	4.14
SPN1.0	0.27	4.01
SPN1.5	0.29	3.78
SPN2.0	0.25	4.38
NPS0	0.20	5.57
NPS0.5	0.23	4.88
NPS1.0	0.27	3.99
NPS2.0	0.22	5.03
NSP0	0.26	4.13
NSP0.2	0.27	4.07
NSP0.4	0.27	4.06
NSP0.8	0.25	4.38

**Table 12:** Results of resistance to abrasion based on the underwater steel ball test method [20].

Mix Number	ARS	Abrasion Rate	AAD
	h/(kg/m^2^)	%	mm
C	5.04	6	9.29
SPN0	6.28	4.7	6.89
SPN0.5	6.44	4.5	6
SPN1.0	6.79	4.3	5.5
SPN1.5	7.38	4	4.86
SPN2.0	7.07	4.1	5.83
NPS0	5.85	5.1	7.87
NPS0.5	5.99	4.9	6.93
NPS1.0	6.97	4.2	5.74
NPS2.0	5.99	4.8	7.58
NSP0	6.7	4.4	6.15
NSP0.2	6.97	4.3	5.77
NSP0.4	7.27	4.1	5.24
NSP0.8	6.97	4.2	5.91

## Data Availability

The data presented in this study are available on request from the corresponding author.
